# Attributes of the food and physical activity built environments from the Southern Cone of Latin America

**DOI:** 10.1038/s41597-021-01073-9

**Published:** 2021-11-01

**Authors:** Laura E. Gutierrez, Natalia Elorriaga, Luz Gibbons, Santiago Melendi, Martín Chaparro, Matías Calandrelli, Fernando Lanas, Nora Mores, Jacqueline Ponzo, Rosana Poggio, Mabel Berrueta, Vilma Irazola

**Affiliations:** 1 South American Center for Cardiovascular Health, Department of Research in Chronic Diseases, Institute for Clinical Effectiveness and Health Policy (IECS), Buenos Aires, Argentina; 2 Centro de Investigación en Epidemiología y Salud Pública (CIESP – CONICET), Buenos Aires, Argentina; 3 Escuela de Nutrición, Facultad de Medicina, Universidad de Buenos Aires (UBA), Buenos Aires, Argentina; 4 Departamento de Salud, Universidad Nacional de La Matanza (UNLaM), San Justo, Buenos Aires, Argentina; 5 Statistics, Data management and Information Systems Unit. Institute for Clinical Effectiveness and Health Policy (IECS), Buenos Aires, Argentina; 6 Sanatorio San Carlos, San Carlos de Bariloche, Argentina; 7 CIGES, Universidad de La Frontera, Temuco, Chile; 8 Municipalidad de Marcos Paz, Buenos Aires, Argentina; 9 Facultad de Medicina, Universidad de la República, Centro Cívico Salvador Allende, Canelones, Uruguay

**Keywords:** Risk factors, Databases

## Abstract

Previous studies have shown the influence of the physical and social environments on the development of obesity and non-communicable diseases (NCD). An obesogenic environment promotes higher dietary energy intakes and sedentary behaviors while limiting opportunities or incentives for active living. This paper presents a dataset with key attributes of the food and physical activity built environment, including green spaces, quality of streets and sidewalks, and different types of food retail outlets in four cities of the Southern Cone of Latin America. A total of 139 representative neighborhoods randomly selected from: Marcos Paz and Bariloche (Argentina), Temuco (Chile) and Canelones-Barros Blancos (Uruguay) were evaluated, where standardized community walks were conducted for direct observation of the built environment. This dataset will contribute valuable data to the evaluation of obesogenic environments in the region, and could be linked to additional ecological information about risk factors for NCDs and socio-economic features from other sources. Understanding environmental influences on cardiovascular risk factors and individual habits may help explain NCD outcomes and plan urban policies.

## Abstract (summary)

**Table:** 

Measurement(s)	features of the built environment
Technology Type(s)	Observation
Sample Characteristic - Environment	built environment
Sample Characteristic - Location	Argentina • Chile • Uruguay

Machine-accessible metadata file describing the reported data: 10.6084/m9.figshare.14892147

## Background & Summary

While obesity and non-communicable disease (NCD) prevention efforts to date have mainly focused on individual health-related behaviors, there is growing recognition of the influence of the physical and social environments in which people live^1–6^. An obesogenic, i.e. obesity-promoting, environment is one that promotes higher dietary energy intakes and sedentary behaviors while limiting opportunities or incentives for healthy food consumption and active living, including active transport and leisure time physical activity^7–13^.

Several studies have shown the relation between characteristics of built environments, from neighborhoods to cities, and prevalence of NCDs and their risk factors, such as obesity, physical inactivity, hypertension and diabetes^14–20^. In addition, some studies suggest that the aforementioned relationships may be more complex than expected^18,21^, be influenced by other related variables^19,22^, and differed across subgroups of the population^23^. Environmental factors and policies affect large segments of the population on a daily basis, and their influence tends to be unevenly distributed across different subgroups. Understanding environmental influences may help to explain healthy and unhealthy behaviors as well as health outcomes^2^.

We conducted a population-based study, which included the systematic observation of a random sample of neighborhoods in four cities from the Southern Cone of Latin America: Marcos Paz y Bariloche (Argentina), Temuco (Chile) and Pando-Barros Blancos-Colonia Nicolich (Uruguay). Representative community areas or neighborhoods were randomly selected to conduct standardized community walks for direct observation of the built environment by trained observers. Data were collected using the Research Electronic Data Capture (REDCap) system in a mobile app on Android tablets.

In this paper, we present the study dataset with food and physical activity attributes of the built environment, including green spaces, quality of the streets and sidewalks, and different types of food retail outlets.

This dataset can be used to characterize the built environment in different urban settings in the Southern Cone of Latin America, including features of typical neighborhoods that may positively or negatively affect walkability such as characteristics of the streets, sidewalks and green spaces. Also, presence and characteristics of food outlets and restaurants can be used to describe key components of the food environment in each location.

Additionally, this dataset represents a valuable opportunity for further analysis, including the use of rigorously measured environmental variables linked to additional ecological information that can be obtained from other sources. For example, there are publically available data from national risk factor surveys, household expenditures and school health databases that may be linked to this database to address research questions on the relationship of the built environment with risk factors for NCDs, socio-economic features or childhood habits (National Institute of Statistics and Censuses from Argentina: https://www.indec.gob.ar/indec/web/Institucional-Indec-BasesDeDatos, National Institute of Statistics from Chile: https://www.ine.cl/estadisticas/ and National Institute of Statistics from Uruguay: http://www.ine.gub.uy/). This way, the relationship between new variables from other sources and characteristics of the built food and physical environment can be explored. This would contribute highly to a more comprehensive evaluation of potentially obesogenic environments in South America, as well as provide input from the region for potential global analyses.

Additionally, further measurements using the same instruments may allow the evaluation of changes over time and may help a deeper understanding of trends in environmental key features and potential recommendations to improve the built environment in our region.

## Methods

### Population and sampling

The neighborhoods included in this study were randomly selected by using the sampling design derived from the CESCAS I study^6^. It used a multistage random sampling strategy in which the first stage consisted of randomly sampling census radii from each location, stratified by socio-economic level. In the second stage, a number of blocks proportional to the radius size were randomly selected. We included all the selected blocks to conduct the community observation walks. Details of the study design of the CESCAS I study have been published elsewhere^24^. Briefly, the study included four small to mid-sized cities in the Southern Cone of Latin America: two cities located in Argentina (Bariloche and Marcos Paz), one in Chile (Temuco), and one in Uruguay (Pando-Barros Blancos-Colonia Nicolich). Marcos Paz and Pando-Barros Blancos are small cities with 54,000 and 58,000 residents, respectively, according to the latest census data. Bariloche (Argentina) and Temuco (Chile) are mid-sized cities with 134,000 and 245,000 residents, respectively, according to the latest census data. These study locations were selected based on population characteristics reflecting the country averages. A total of 139 representative community areas or neighborhoods were randomly selected to conduct standardized community walks with direct observation of the built environment (23 in Marcos Paz; 25 in Bariloche; 48 in Temuco and 43 in Canelones).

### Data collection

**Fig. 1:**
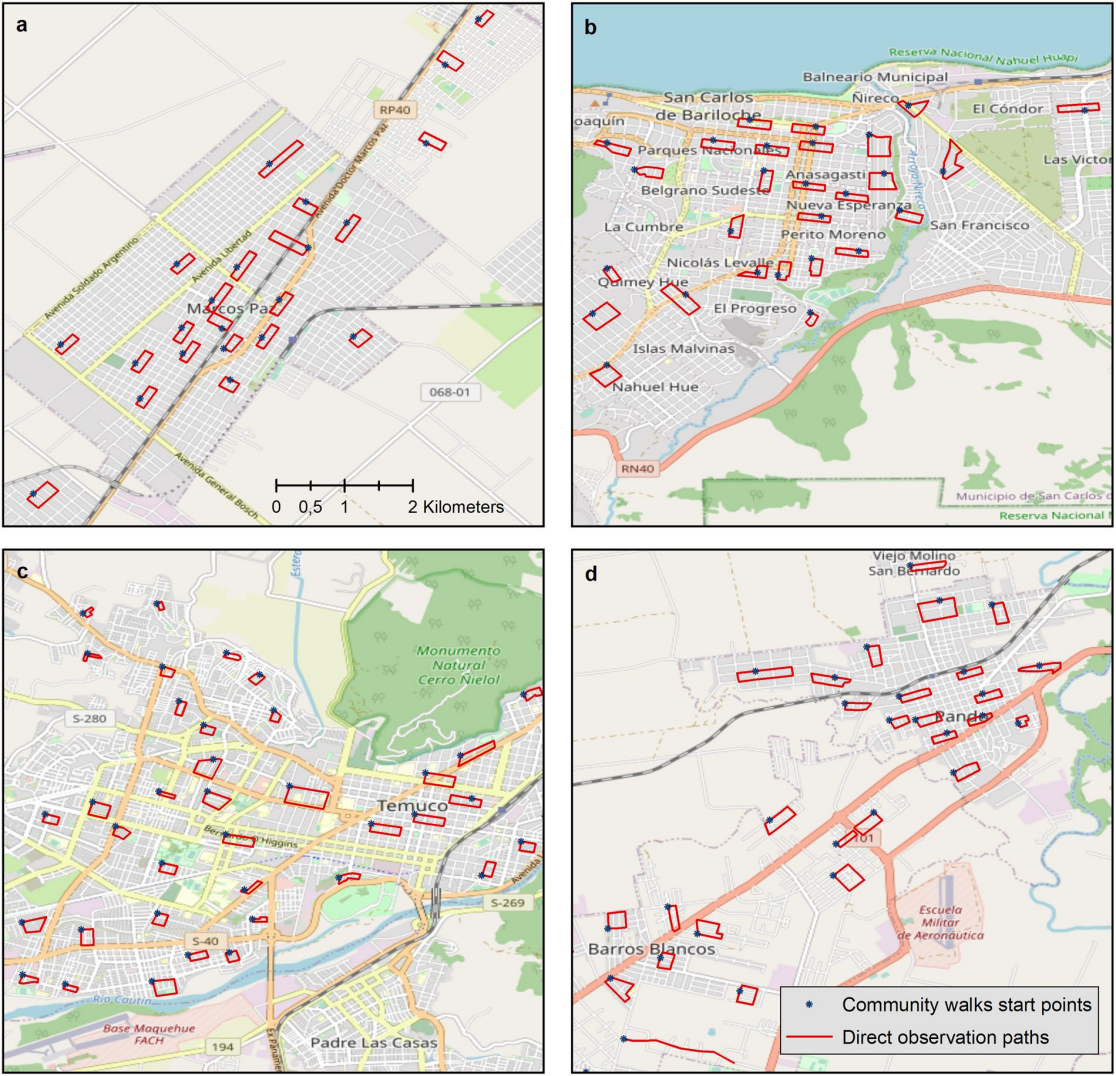
Geographical distribution of observation walks in the study sites. (**a**) Marcos Paz, Argentina; (**b**) Bariloche, Argentina; (**c**) Temuco, Chile and, (**d**) Pando and Barros Blancos, Uruguay. All maps are on the same scale (1:40.000). Coordinate System: WGS1984. Base map layer credits: © OpenStreetMap contributors (https://www.openstreetmap.org/copyright/en).

Trained and certified observers conducted all the community observation walks to assess the built physical and food environment of the neighborhoods. Within each community observation walk, trained observers collected data on selected attributes of the built environment, including quality of the streets and sidewalks, street infrastructure (lighting, crosswalk and traffic light) and food retail outlets and restaurants. In each selected neighborhood, a random start point was determined, from where observers completed an eight-block route by following a clockwise direction (Fig. 1). All the observations were performed during daylight in concurred hours. Observers had an explicit route and a map of the surroundings.

Because in the four cities included in the study, the native language is Spanish, translated and adapted versions of previously validated instruments were used.

To evaluate the streets, sidewalks and surrounding, selected questions from the EPOCH-1 instrument^25^ were used. To characterize the food stores and restaurants, we selected items from NEMS-S^26^ and EPOCH-1^25^. Finally, the POS Tool^27^ was used to describe the public open spaces.

All data management activities were led by the Data Coordinating Center (DCC) at IECS. Data collection was performed using Research Electronic Data Capture (REDCap) system in a mobile app on Android tablets.

REDCap is a web-based software developed at the Centre for Clinical and Translational Research at Vanderbilt University in Nashville, Tennessee, USA. REDCap provides online and offline users with the ability to create and manage clinical research databases in compliance with Good Clinical Practice^28,29^. The REDCap Mobile App (offline data collection) is a mobile application that was run off-line on tablets.

During the community observation walks, data were collected directly in REDCap using tablet devices. The tablets used in the field required double permission to access the system, first to login on the device and then a REDCap account with a 6-digit pin were required to access the application. At end of the community observation walk, tablets were synchronized via an internet connection and data were uploaded into the study database. Access to the data was restricted by study site.

All variables were captured in electronic forms designed for this study. The system allowed real-time data entry validation by specifying a Field Validation or Data Quality Rule, which improved data integrity by avoiding errors before data were saved to the database. The app also used a branching logic that allowed for showing or hiding fields depending on the value input of previous fields.

After the data were entered and uploaded in REDCap, additional process validation were used to ensure data were complete, consistent, logical, and accurate. External rules to detect missing data, out of range values and inconsistent data were defined and programmed using SAS 9.3. Quality checks based on those rules were run weekly, and a spreadsheet was generated with all the discrepancies found. These discrepancies were resolved by investigators after logging into the system. Additionally, reports with plots and descriptive statistics for each variable were generated monthly in the module “Stats & Charts” of REDCap. These reports contained absolute and percent values for category variables. For continuous variables, these reports included percentage of missing values, minimum and maximum values, mean, standard deviation and percentiles.

Quality control measurements reflected ongoing, day-to-day review of data generated from approved protocol-driven procedures. The DCC supported weekly on-site monitoring by Site Field Coordinators. Weekly phone calls were carried out to monitor fieldwork processes and data quality. Electronic forms were checked for completeness, data accuracy, and correct storage in a weekly basis. Further, data quality and monitoring reports were generated weekly from the REDCap system by the DCC and sent to Principal Investigators and Co-investigators.

Datasets were kept inside REDCap, where they were safe, available for look-up, and logged according to Good Clinical Practice (GCP). Data was exported in SAS format and placed in a secure Dropbox folder. Additionally, for long-term preservation, the database and code labels were stored as delimited.

## Data Records

The dataset, dictionary, CRFs, protocol of the study and metadata document are hosted on the Mendeley repository^30^. Table 1 supplies details of each available file.

**Table 1:** List of files in the repository.

Files	Name	File description	Format
Metadata	Obesogenic environment observation_Metadata	Provides information of each available file.	csv
Dataset	Obesogenic environment observation_Dataset	Contains the variables collected to describe features of the built environment, including quality of the streets and sidewalks, street infrastructure, different types of food retail outlets and restaurants, and public open space. Each record contains the data about one community observation walk.	csv
Data dictionary	Data Dictionary	Describes the attributes of the variables included in the dataset.	csv
Case report Form 1: Observation of the environment	CRF 1. Environment Observation	Instrument used to register data from the observation of the environment.	pdf
Case report Form 2: Observation of the public open space	CRF 2. Open Public Space Observation	Instrument used to register data from public open spaces.	pdf
Protocol	Protocol_Food and physical activity built environments in the Southern Cone of Latin America	Describes the objectives, design, methodology and organization of the study	pdf

## Technical Validation

The technical validation and quality control of the data were conducted following three steps. The first step was carried out before the fieldwork and consisted of the development of procedures for standardized data collection and operation manuals. The second step encompassed training workshops and certification of the data collectors and pilot testing in the field. The third step took place during the fieldwork and included both supervision of data collection in the field and remote quality checks in real time. For this process, each GPS location marked by the observer in the field was retrieved from REDCap and validated; guaranteeing that the data collected matched the correct geographical area. These GPS locations included the starting point for each community observation walk, as well as the location of food stores, restaurants and public open spaces. Key aspects of the instruments were centrally supervised, and data collected by observers were backed-up with on-site photos taken as part of the direct observation walks. Additionally, Google Street View was used to supervise and validate all collected data^31^. This tool is available from Google’s online Maps application (http://maps.google.com) and displays street level panoramas of the cities around the world. Queries were generated and sent to the observer for resolution when any of the following conditions were met: (a) Distance of the collected GPS coordinates differed from the start point assigned, (b) On-site photos or Street View images differ from the data collected, (c) Inconsistencies within the data collected, (d) Out of range values, and (e) Missing values.

## Data Availability

No custom code was used in this study.
